# Unmasking vaccine hesitancy and refusal: a deep dive into Anti-vaxxer perspectives on COVID-19 in Spain

**DOI:** 10.1186/s12889-024-18864-5

**Published:** 2024-07-01

**Authors:** Ángela Prieto-Campo, Olalla Vázquez-Cancela, Fátima Roque, Maria Teresa Herdeiro, Adolfo Figueiras, Maruxa Zapata-Cachafeiro

**Affiliations:** 1 Department of Public Health, Faculty of Pharmacy, University of Santiago de Compostela, Santiago de Compostela, A Coruña, Spain; 2 Department of Preventive Medicine, Santiago de Compostela University Teaching Hospital, Santiago de Compostela, A Coruña, Spain; 3 Research Unit for Inland Development (Unidade para o Desenvolvimento do Interior/UDI-IPG), Polytechnic of Guarda, Guarda, Portugal; 4 Health Sciences Research Centre (Centro de Investigação em Ciências da Saúde/CICS-UBI), University of Beira Interior, Covilhã, Portugal; 5 School of Health Sciences (Escola Superior de Saúde/ESS), Guarda Polytechnic Institute, Guarda, Portugal; 6 Department of Medical Sciences, Institute of Biomedicine (iBiMED), University of Aveiro, Aveiro, Portugal; 7 Consortium for Biomedical Research in Epidemiology and Public Health (CIBER en Epidemiología y Salud Pública/CIBERESP), Carlos III Institute of Health, Madrid, Spain; 8 Health Research Institute of Santiago de Compostela (Instituto de Investigación Sanitaria de Santiago de Compostela/IDIS), A Coruña, Spain

**Keywords:** Vaccination refusal, COVID-19, Knowledge, perception

## Abstract

**Background:**

At the time of the emergence of COVID-19, denialist and anti-vaccine groups have also emerged and are shaking public confidence in vaccination.

**Methods:**

A qualitative study was conducted using online focus groups. Participants had not received any doses of vaccination against the disease. A total of five focus group sessions were conducted with 28 participants. They were recruited by snowball sampling and by convenience sampling.

**Results:**

The two major topics mentioned by the participants were adverse effects and information. The adverse effects described were severe and included sudden death. In the case of information, participants reported: (1) consultation of websites on which scientists posted anti-vaccination content; and (2) distrust.

**Conclusions:**

At a time when anti-vaccine groups pose a major challenge to public health in general, and to COVID-19 vaccination campaigns in particular, this study is a first step towards gaining deeper insight into the factors that lead to COVID-19 vaccine refusal.

**Supplementary Information:**

The online version contains supplementary material available at 10.1186/s12889-024-18864-5.

## Introduction

### The role of vaccines in the COVID-19 pandemic

In the more than 2 years that have elapsed since it first appeared, SARS-CoV-2 has spread uncontrollably around the world [1]. In all this time, it has caused a progressive rise in morbidity, mortality (direct and indirect), health service overloads, and financial problems [2]. To date, in the absence of specific treatments to combat the disease, the development of vaccines has proved to be the most important public health strategy for tackling COVID-19. Since the start of vaccination, there has been a dramatic reduction in cases, hospitalisations and deaths [3].

### The reality of vaccination coverages

That said, however, COVID-19 vaccination uptake was far from ideal and is steadily declining with each successive dose and booster required by the disease’s evolution. According to the European Centre for Disease Prevention and Control, while 73% of the European population received the primary course of vaccination, only 54,8% had the first booster and a mere 6.7% the second booster [4]. It was thus of little use to have vaccines available, if the vaccination programme against the disease is not effective. Success of vaccination campaigns relied on vaccination uptake being high in all doses and equitable across the globe, something that, in turn, depended on there being adequate access and delivery in all countries. By way of a further indispensable condition, acceptance by the target population is of the essence [5].

### Reasons behind vaccination hesitancy and refusal

Lack of acceptance may have arisen for two main reasons: (1) because the candidates for vaccination were in a state of vaccine hesitancy; or alternatively (2), because they displayed a radical anti-vaccination attitude linked to science denialism in general (climate change negationists, flat earthers, etc.) [6–9]. Hesitant individuals were beset by indecision and uncertainty when it came to taking the vaccination decision, due to doubts about vaccine safety, vaccine efficacy, the desire to feel freedom and the right to be able to freely choose whether or not to vaccinate without social constraints; concerns about the false link between vaccination and diseases such as autism, based on discredited scientific studies; conspiracy theories advocating the existence of a population control plan through the implantation of chips and genetic manipulation; lack of trust in health professionals, health authorities, or government; the perceived low risk and low awareness of the benefits of vaccination due to the success of vaccination programmes; the belief that natural immunity is always better and more durable than that acquired through vaccination; the misinformation they receive from anti-vaccine groups and anti-science in general; financial interests on the part of pharmaceutical companies; government and even health professionals; religious beliefs and the associated health decisions they entail [8, 10–16]. This moment in time represented the intermediate point between having a pro-vaccination or an anti-vaccination stance [6]. This is why vaccine hesitancy could lead to anti-vaccination behaviour, generally as a consequence of the action of anti-vaccine (antivaxxer) groups. These were very well-defined groups, with financial or political purposes, that took advantage of moments of vulnerability by disseminating misinformation against vaccines over the social media, or discrediting scientific studies [17]. In this way, they managed to influence the vaccination behaviour of the vaccine-hesitant, shifting them from vaccine-hesitancy to anti-vaccination status [15].

### Need for a qualitative approach

Qualitative research makes it possible to carry out an in-depth examination of the reasons (beliefs and perceptions, fears and attitudes) for the most complex human behaviours (including the vaccination decision), something that with any other methodology would be more complicated [18]. Interaction among participants can bring forth ideas and beliefs unknown to the researcher and, sometimes, even to the individuals under study [19, 20]. Qualitative research can thus be a very useful tool in some fields of biomedical research, such as vaccine hesitancy [21]. The aim of this study was therefore to ascertain the knowledge, beliefs, attitudes and barriers of individuals who had not been vaccinated of their own volition and belonged to anti-vaccine groups.

## Methods

### Study setting and study-design

The study was conducted throughout Spain, a country of 47 million inhabitants [22]. In Spain, vaccination rate against infectious diseases is generally high, but the optimal and desired vaccination rate is never achieved [23]. In this case, the figures shows high but not full vaccination coverage against COVID-19 [24]. In fact, when this study began, 4.5 million people had not yet received any dose of the vaccine [25].

We carried out a qualitative study. The focus group (FG) method was used to explore antivaxxers’ knowledge, perceptions and barriers regarding COVID-19 vaccines. We decided to use the FG technique so that interaction between group members could take place and all dimensions of the problem could then be addressed by obtaining different points of view [26]. In addition, FG sessions were held online because, compared to the traditional face-to-face method, they are less costly, allow for the inclusion of people who are geographically far apart, prevent the possibility of COVID-19 transmission among participants, are easier to organise, and are also more convenient for the participants [27]. The **CO**nsolidated criteria for **RE**porting **Q**ualitative research (COREQ) were applied [28].

### Selection, sample and procedure

Antivaxxers were recruited by snowball sampling and by convenience sampling on the Telegram social network from groups with anti-vaccine content. To this end, we sent a message with an explanation of the study and a proposal to participate. As a next step, subjects who decided to participate were sent a link to select their availability to participate in the study. We then formed groups of participants and set the dates according to their availability in the order of acceptance to participate in the study. Once the dates had been set, focus group participants were then sent a reminder and a link to the online meeting, the day before. Participants from 20 groups were contacted, and finally 28 individuals participated in 5 online FG (Table 1). The FG meetings were held in February and March 2022.

**Table 1:** Demographic characteristics of focus group participants

Characteristics	Group 1 (*n* = 3)	Group 2 (*n* = 6)	Group 3 (*n* = 5)	Group 4 (*n* = 6)	Group 5 (*n* = 8)
Age					
Mean (SD)	32.7(2.5)	43.5(10.7)	30.4(3.4)	37.7(2.9)	40.8(6.2)
Sex, No. (%)					
Female	0 (0.0)	4(66.7)	3(60.0)	4(66.7)	6(75.0)
Male	3(100.0)	2(33.3)	2(40.0)	2(33.3)	2(25.0)
Area of residence, No. (%)					
Urban	3(100.0)	4(66.7)	4(80.0)	4(66.7)	7(87.5)
Rural	0(0.0)	2(33.3)	1(20.0)	2(33.3)	1(12.5)

We developed a script for use in the FG based on literature on vaccine hesitancy and vaccine refusal published to date [29–31]. For this purpose, we were also assisted by a group of epidemiology and pharmacology experts, as well as a psychologist (AF, MZC, ALD).

The FG sessions were guided by two researchers (AP and OV). At the end of each session, a checklist (Supplementay Material 1) was used to verify whether all the topics of interest in the script had been addressed. In this way, we determined when information saturation was reached. The sessions were conducted through the Microsoft Teams platform, and all sessions were recorded after a warning had been issued to this effect, though all recordings were deleted after analysis. Participants had the option of appearing in the FG with the camera activated or not. The sessions lasted 60 to 90 min, and ended when the participants contributed no new ideas. AP made the literal transcriptions, listening to the same recording again 2 days later to check that it had been correctly transcribed. Participants were coded with numbers according to the FG to which they belonged and the number of participants in each group (FG1P1, FG2P2, etc.).

### Ethical considerations

This study was submitted for evaluation by the Galician Clinical Research Ethics Committee *(Comité de Ética de Investigación de Galicia)*, which assessed it and declared that no approval was necessary given its nature. All study participants were informed of the purpose and objectives of the study (prior to and during the sessions), and that the FG sessions would be recorded and transcribed without participants being personally identified in the study results. All participants gave express oral consent to participate prior to the start of the FG recording.

### Analysis

Thematic analysis was used. This is a method for analysing qualitative data which involves interpretation in the processes of selecting codes and constructing topics [32]. AP repeatedly read the transcripts until she became thoroughly familiar with the contents, and then started to take notes on possible elements of interest and generate initial codes with the most basic information. The topics were then constructed by analysing or combining the initial codes. Lastly, AF and MZC reviewed the appropriateness of the topics created, and these were defined and described. No computer software programme was used to analyse the process.

## Results

5 FG were formed with a total of 28 participants. The general characteristics of participants are described in Table 1. Seven topics and 24 subtopics were identified after transcription and analysis. The topics and subtopics identified along with the FGin which they emerged are listed in Table 2. Example quotes are shown in Table 3.

**Table 2:** Thematic analysis topics, subtopics, and focus groups in which they emerged

TOPIC	SUBTOPIC	FG1	FG2	FG3	FG4	FG5	QUOTES
Virus and disease	*Non-existence*	✓	✓	✓	✓	✓	29
	*Relatively unimportant disease*	✓	✓	✓	✓	✓	16
Vaccine	*Not a vaccine*	✓	✓	✓	✓	✓	28
	*Composition*	✓	✓	✓	✓	✓	50
	*Does not immunise*	✓	✓	✓	✓	✓	30
	*Adverse effects*	✓	✓	✓	✓	✓	85
	*Benefits*	✓	✓	✓	✓	✓	16
	*Differences between vaccines*	✓	✓	✓		✓	8
	*Consent and medical prescription*	✓	✓			✓	10
	*Vaccination in children*	✓	✓				5
Information	*Inadequate*	✓	✓	✓	✓	✓	41
	*More than adequate*	✓	✓	✓	✓	✓	19
	*Sources of information*	✓	✓	✓	✓	✓	68
Distrust	*Mass media*	✓	✓	✓	✓	✓	22
	*Globalist families*	✓	✓	✓	✓		16
	*Pharmaceutical industry*	✓	✓	✓		✓	13
	*Politicians*	✓	✓	✓	✓	✓	24
	*Health professionals*	✓	✓	✓	✓	✓	30
Conspiracies	*Reduction of the world population*		✓	✓	✓	✓	7
	*Population control*	✓	✓	✓			9
	*Previously programmed situation*	✓		✓	✓	✓	6
Other vaccines	*Different*	✓	✓			✓	18
	*Refusal*	✓	✓	✓	✓	✓	23
Influences	*Pressure*	✓	✓	✓	✓	✓	9

**Table 3:** Thematic analysis topics, subtopics, and example quotes

TOPIC	SUBTOPIC	QUOTES
1. Virus and disease	*1.1Non-existence*	1.1.1.[“*I don’t believe there’s any virus”.*] (FG1P2). 1.1.2.[*“The Spanish government has said that it doesn’t have the cultures, and their existence has never really been proved […] So, if no virus has been shown to exist, how can there be a vaccine for a non-existent virus? “*] (FG2P4). 1.1.3.[*“Where are the dead bodies on the pavement covered by a sheet? That’s a pandemic!”*] (FG4P3). 1.1.4.[“*They immediately label you a paranoid conspiracy theorist, a negationist”.*] (FG3P1).
	*1.2Relatively unimportant disease*	1.2.1.[“*Smoking kills more people than this supposed virus.”*] (FG1P2). 1.2.2.[“*What’s happening is simply what’s always bloody happened, when there was ’flu.”*] (FG2P1). 1.2.3.[“*I haven’t been sick in 2 years and I always go around without a mask and I mix with a lot of people.”*] (FG3P1). 1.2.4.[“*Not everything is COVID. There are other diseases and they have to be treated.”*] (FG4P5).
2. Vaccine	*2.1. Not a vaccine*	2.1.1.[*“The fact is they’re not vaccines. To begin with, they’re not vaccines. They’re inoculations.”*] (FG2P1). 2.1.2.[“*I’m against pseudovaccines.”*] (FG1P1). 2.1.3.[“*Something that’s not proved and is in an experimental phase can’t be said to be the panacea.”*] (FG4P5). 2.1.4.[“*It’s an experimental drug that’s in phase II, which is a clinical trial.”*](FG3P1).
	*2.2. Composition*	2.2.1.[“*You go home and you’ve no idea of what they’ve given you.”*] (FG5P3). 2.2.2.[*“The problem is not the vaccine itself, the problem is this inoculation which carries messenger RNA.”*] (FG2P2). *2.2.3.*[*“There are vials with different compositions and forms of attack, because uniformity in attack would mean there’s a cause-effect relationship between the vaccine and the subsequent effect.”*] *(FG1P2).*
	*2.3. Alternative therapies*	2.3.1.[“*The treatment’s no good. It doesn’t prevent you from getting infected or infecting others.”*] (FG4P3). 2.3.2.[*“People take all the jabs, up to the third one, but they keep on falling ill and infecting others.”*] (FG5P5). 2.3.3.[“*I get vaccinated for my own good not for others’.”*] (FG2P1). 2.3.4.[“*Eat healthily and do exercise.”]* (FG1P1) 2.3.5.[“*For example, the Zelenko method […], they’re treatments to boost your defenses […] with many natural things, in general these are vitamin D, vitamin C, zinc, magnesium…”*](FG1P3). 2.3.6.[“*I’ve always been a fan of herbs, the most natural and traditional medicine.”*] (FG3P2).
	*2.4. Adverse effects*	2.4.1.[“*It seems to me like a heart-attack epidemic right now.”*] (FG3P5). 2.4.2. [“*Cases of myocarditis have shot up by 400–500%.”*] (FG3P3). 2.4.3.[“*There are more and more cases of stroke appearing.”*] (FG5P7). *2.4.4*.[*“There are top international football, basketball players… with heart attacks or stroke.”*] (FG3P3). 2.4.5.[“*The husband of a friend got vaccinated on Saturday and died on Sunday.”*] (FG4P5). 2.4.6.[“*It’ll affect some people in one way and other people in another.”*] (FG2P1). *2.4.7.[“Traditional vaccines that are tested, still cause autism in some cases …”]* (FG1P1). 2.4.8.[“*And you go home, and in a year’s time, you’ve no idea of the consequences, in the long term, the medium term or the short term.”*] (FG5P3). 2.4.9.[“*Adverse effects? If, what you choose to call adverse or side-effects are what I believe to be the primary or direct effects”*] (FG1P1).
	*2.5. Benefits*	2.5.1.[“*It has the advantage of a COVID passport, or of being able to travel, or of greater freedom.”*] (FG1P3).
	*2.6. Differences between vaccines*	2.6.1.[“*If I offered you cyanide made by Iván or made by Feli, or made by Olalla… with which cyanide would you want to poison yourself? “*](FG2P6). 2.6.2.[*“The highest mortality has been recorded by Moderna and Pfizer, which are the ones that use messenger RNA techniques.”*] (FG3P1).
	*2.7. Consent and medical prescription*	2.7.1.[“*Anything that’s intravenous or intramuscular needs a medical prescription.”] (FG5P1).* 2.7.2.[“*All vaccines need a medical prescription and informed consent”*] (FG5P1).
	*2.8. Vaccination in children*	2.8.1.[“*In everybody it’s a mistake, but in children it’s terrible. “*] (FG1P2)
3. Information	*3.1. Inadequate*	3.1.1.[“*Médicos por la Verdad [Doctors for Truth] is a very powerful organisation that is highly censored.”*] (FG3P3). 3.1.2.[“*First, we began with the claim that it gave you 90% protection, then 70% protection, then that you’d have fewer symptoms, and afterwards that you’d avoid being put in an ICU.”*] (FG1P1). 3.1.3.[*“At the beginning (…) you had to have 30% of the population vaccinated. When we got to 30% it went to 50%, when 50% of the population had been vaccinated, it went to 70%, and now we’ve reached 90%, and that’s still not enough.”*] (FG1P3). 3.1.4.[“*What a coincidence that previously they neither became infected nor developed symptoms or infected others, but now all of a sudden they’re the most vulnerable group. So where does that leave us?“*] (FG1P1).
	*3.2. More than adequate*	*3.2.1.[“Those of us who are here are in the habit of checking information, and that’s why we’re not vaccinated.”*] (FG5P1).
	*3.3. Sources of information*	3.3.1.[“*There are lots of scientists, doctors, and super qualified people who don’t agree with vaccines.”*] (FG3P1). *3.3.2.[“I’m in (Telegram) anti-vaccine groups of doctors, therapists and others.”]* (FG4P4). 3.3.3.[*“You don’t know who to believe, to believe the TV news, to believe a doctor who takes orders from the Ministry of Health.*”] (FG5P3).
4. Distrust	*4.1. Mass media*	4.1.1.[“*I haven’t seen that particular version of what they’re telling me. On the TV I saw stretchers, dead people, the end of the world… but, hang on a sec, in my immediate surroundings, in my world, I don’t see that”*] (FG4P3).
	*4.2. Globalist families*	4.2.1.[*“They can do absolutely whatever they want, like Bill Gates with his vaccines in Africa”*] (FG1P3).
	*4.3. Pharmaceutical industry*	4.3.1.[“*I think that if, despite this mass propaganda campaign (in the media) they still haven’t been vaccinated, it’s because they really don’t want to get vaccinated”*] (FG1P3).
	*4.4. Politicians*	4.4.1.[“*All the information that comes from the Ministry of Health, I put in quarantine.”]* (FG4P2). 4.4.2. *[“When a politician (…) starts acting and talking like a health professional…that smells bad to me.”]* (FG5P1).
	*4.5. Health professionals*	4.5.1.[“*They’d have to go around wearing a white lab coat, but like Formula 1 cars, with the names of their sponsors written on it.”*] (FG1P1). 4.5.2.[“*They charge for COVID hospitalisation, they charge for COVID ICUs, and they charge for COVID death.”*] (FG2P6). 4.5.3.[“*They’re not interested in people’s health, they’re interested in making diseases chronic and doing business.”*] (FG4P4). 4.5.4.[“*I think we’re going to lose a lot of trust in doctors, in our present doctors, the ones who also wanted to shove this blessed vaccine down our throats.”*] (FG1P3).
5. Conspiracies	*5.1. Reduction of the world population*	5.1.1.[“*It’s been developed and planned by all sectors, by the mass media, by the politicians, by the health sector.”*] (FG4P1). 5.1.2.[“*The want the human race, as we know it, to disappear, and their brains to be monitored.”*] (FG5P3). 5.1.3.[“*The first cases of COVID appear with the 2019 influenza vaccine. Curiously, almost all the elderly in nursing homes were vaccinated and they were the first to drop.”*] (FG2P6).
	*5.2. Population control*	5.2.1.[*“They cover it up and disguise it with new strains to fool the herd, and that’s that.”*] (FG5P3).
	*5.3. Previously programmed situation*	5.3.1.[“*It’s been developed and planned.”] (FG4P1).*
6. Other vaccines	*6.1. Different*	6.1.2.[“*A polio vaccine, for instance, doesn’t help you to have fewer effects of polio; from the moment go it protects you completely …and that, even according to the official narrative, doesn’t protect you 100%.”*] (FG5P7). 6.1.3.[“*The ten years that are needed (to develop a vaccine) …. The 10 years can’t be bought.”*] (FG2P1). 6.1.4.[“*We have no problem with vaccines, what we have a problem with are mRNA inoculations.”*] (FG2P2).
7. Influences	*7.1. Pressure*	7.1.1.[“*There’s only one way they’re going to vaccinate me, the Guardia Civil* [Spain’s paramilitary police force] *will have to come and fetch me. And we’re not far away from that with the pressure they’re putting us under… They’ll have to drag me away”.*] (FG2P1).

### Virus and disease

#### Non-existence

All FG expressed doubts about the **existence** of SARS-CoV-2, and the reason most cited was the lack of **isolation or sequencing** of the virus (quote 1.1.1.). Some participants even referred to other countries to which they had travelled, in which there was no virus (quote 1.1.2.). Similarly, 4 groups also denied that we might ever have gone through a **pandemic** situation [“*Where are the dead bodies on the pavement covered by a sheet? That’s a pandemic!”* (quote 1.1.3)]. Despite affirming that neither the virus nor the disease existed, the participants from 3 groups nevertheless complained of being considered **negationists** (quote 1.1.4.).

#### Relatively unimportant disease

COVID-19 was considered somewhat irrelevant by unvaccinated individuals, who commented that it was a **relatively mild** illness (quote 1.2.1.) and similar to seasonal **influenza** (quote 1.2.2.). In addition, three participants denied the **contagiousness** of the disease, based on their experience of not using a face mask and not having contracted the disease (quote 1.2.3.). Two groups mentioned that far too much attention was being paid to COVID-19 and **other higher-priority diseases were being neglected** (quote 1.2.4.).

### Vaccine

#### Not a vaccine

None of the participants would condescend to give vaccine status to COVID-19 vaccines, and instead repeatedly referred to them as “**inoculations”, “jabs”, “injections”** or **“pseudovaccine”** (quote 2.1.1.); (quote 2.1.2.).

Most of them commented that the vaccines were **experimental**, since they had not gone through all the required phases (quote 2.1.3.), and in one group, some subjects described the vaccination process as part of a **clinical trial** (quote 2.1.4).

#### Composition

One of the factors of most concern to the unvaccinated was the composition of the vaccines, since many felt uneasy about **not knowing their content** (quote 2.2.1.). Among the different components cited by the participants as being known and dangerous (graphene oxide, heavy metals, poison, carcinogens, etc.), special mention should be made of the importance attached to **mRNA** (quote 2.2.2.), which they do not consider fit for use in vaccines on human beings.

One participant claimed that there were **batches with different compositions**, aimed at giving rise to different effects in each person [“*There are vials with different compositions and forms of attack, because uniformity in attack would mean there’s a cause-effect relationship between the vaccine and the subsequent effect”* (quote 2.2.3)].

#### Does not immunise

The perception that the vaccine **neither immunises** the individual nor prevents contagion despite the **successive doses** administered (quote 2.3.1.) was present in all the groups [*“People take all the jabs, up to the third one, but they keep on falling ill and infecting others”* quote 2.3.2.)]. Furthermore, in two groups, participants acknowledged that **herd immunity** was of no concern to them and that they only cared about their own protection (quote 2.3.3.).

Another two groups proposed leading a **healthy** lifestyle as the right way of achieving immunity (quote 2.3.4.) *and* put their trust in **natural medicine** (quote 2.3.5.); (quote 2.3.6.).

#### Adverse effects

All the participants reported some adverse effect linked to COVID-19 vaccines which had been experienced in their immediate circle. Most of them cited **severe** cardiovascular or thromboembolic effects *[“It seems to me like a heart-attack epidemic right now”* (quote 2.4.1.)]; (quote 2.4.2.); (quote 2.4.3.), especially among **young people and sportspersons** (quote 2.4.4.), though they also mentioned many other important effects, such as immune system disorders, HIV, or even COVID-19. Moreover, all the groups said they had first-hand knowledge of cases of **death** after vaccination, something that they nick-named “*suddenitis* (“*repentinitis*”, quote 2.4.5.). To a lesser extent, participants in 2 groups also talked about **mild** effects like those caused by traditional vaccines, such as high temperature or upper-arm pain.

The idea that was uppermost among unvaccinated individuals was that the vaccine might affect each individual in a different way (quote 2.4.6.), and there was also perceived uncertainty about the effects, which were still unknown and might occur in the **long term** (quote 2.4.7.). Two participants felt that the effects described were not side-effects at all but rather “**direct or primary**” effects (quote 2.4.8.).

#### Benefits

The only benefits of COVID-19 vaccines acknowledged by the participants were **social**, i.e., being able to travel, engage in recreation and leisure activities, and even work. In no case however did they report health benefits (quote 2.5.1.). Other subjects said directly that vaccination brings **no** benefit.

#### Differences between vaccines

The great majority of participants were of the opinion that all the vaccines developed against SARS-CoV-2 were **equally** harmful (quote 2.6.1.); only one of them considered vaccines that use **mRNA** techniques to be more dangerous (quote 2.6.2.).

#### Consent and medical prescription

Three groups reported that vaccination cannot be administered without a physician’s prior **prescription** and the patient’s **written informed consent**(quote 2.7.1.); (quote 2.7.2.).

#### Vaccination in children

Some unvaccinated individuals displayed even greater rejection of vaccines when talking about immunisation in **children** (quote 2.8.1.).

### Information

#### Inadequate

Most of the participants considered that we have scant information about COVID-19 and the vaccination process. On the one hand, there are **few studies** on vaccines, and, on the other, information from experts about the risks of vaccination is being **censored and manipulated** (quote 3.1.1.). Furthermore, official sources make **continuous changes** in the information that they hand out to the public on the protection afforded by vaccine, the percentage vaccination needed to achieve herd immunity, or the characteristics of the disease in children and pregnant women [“*First, we began with the claim that it gave you 90% protection, then 70% protection, then that you’d have fewer symptoms, and afterwards that you’d avoid being put in an ICU”* (quote 3.1.2.)]; (quote 3.1.3.); (quote 3.1.4.).

#### More than adequate

Some participants, in contrast, believed that the **information did indeed exist**, but that people nonetheless consented to be vaccinated without **looking into the matter** and without checking the information, unlike what they themselves did (quote 3.2.1.).

#### Information sources

As a reliable source of information, the great majority of the participants used anti-vaccine scientists or health professionals, whom they considered to be **experts** (quote 3.3.1.). Many of these are in **Telegram** [*“I’m in (Telegram) anti-vaccine groups of doctors, therapists and others”*(quote 3.3.2.)], which is the preferred online platform for seeking and sharing information. Even so, some participants admitted that they did not consider **any** source of information reliable (quote 3.3.3.).

### Distrust

#### Mass media

All the groups considered that the information provided by the mass media did not correspond to reality. The television has manipulated COVID-19 deaths and has bombarded the population with disinformation (quote 4.1.1.).

#### Globalist families

The participants accused certain globalist families (especially Bill Gates’) of **controlling** the world and having **power** over the pharmaceutical industry and politicians, which they use to develop vaccines harmful to the **population** (quote 4.2.1.).

#### Pharmaceutical industry

The participants pointed to the pharmaceutical industry as a multinational corporate entity whose sole interest is to ensure that there are diseases, so that it can **sell** the maximum possible amount of medications (quote 4.3.1.), and it is therefore fully aware of and **complicit in** the adverse effects of the vaccines.

#### Politicians

To people wary of vaccination, the information and indications provided by the government lacked credibility (quote 4.4.1.), and four groups identified the virus as being a **political** rather than a health **issue** (quote 4.4.2.).

#### Health professionals

All the groups made reference to health professionals. The participants were of the opinion that doctors’ medical practices are **profit-driven** (quote 4.5.1.), both in the case of COVID- 19 [*“They’d have to go around wearing a white lab coat, but like Formula 1 cars, with the names of their sponsors written on it.”* (quote 4.5.1.)], and in their routine clinical practice (quote 4.5.3.). Furthermore, the appearance of COVID-19 has only worsened this situation by increasing **distrust** in hospitals [*“I think we’re going to lose a lot of trust in doctors, in our present doctors.”* (quote 4.5.4.)] and health institutions such as the World Health Organisation.

### Conspiracies

In all groups, paranoid conspiracy feelings predominated, e.g., that the aim of the pandemic was to reach the maximum possible number of vaccinated persons, a pre-programmed multidisciplinary **plan** (quote 5.3.1.) aimed at **reducing** the world population (quote 5.1.1) (quote5.1.2.) and **controlling** people (quote 5.2.1.).

### Other vaccines

#### Different

A percentage of the participants considered that vaccines developed prior to COVID-19 are very different (quote 6.1.1.), in that they offer real **protection** [“*A polio vaccine, for instance, doesn’t help you to have fewer effects of polio; from the moment go it protects you completely, and that, even according to the official narrative, doesn’t protect you 100%”* (quote 6.1.2.)], were studied for the required length of **time** (quote 6.1.3.), and contain **inert virus** instead of messenger RNA (quote 6.1.4.).

#### Refusal

Other participants rejected any vaccine, and the main reason was because of the **adverse effects**. Autism in children was the effect most described for all vaccines in general (quote 6.2.1.), but there were many more (bacterial infections, disability, etc.) which were related to specific vaccines, such as human papilloma virus (HPV) or meningitis vaccines. There was even one participant who had the idea that COVID-19 was a side-effect of the influenza vaccine (quote 6.2.2.).

Two groups made the point that the development of vaccines against SARS-CoV-2 is increasing doubts and rejection of traditional vaccines [“*Right now we’re thinking not only about the need for this vaccine, but also about the need for all the others”* (quote 6.2.3.)].

### Influences

The participants felt that there was a great deal of **pressure** exerted by leaders, the mass media, relatives and colleagues for people to be vaccinated. Even so, all the coercion has been in vain (quote 7.1.1.).

## Discussion

### Main results

At a time when denialism of science in general and vaccine efficacy and safety in particular is expanding alarmingly and globally [33, 34], ascertaining the perceptions and attitudes of negationists to vaccination may be of great interest for public health. To our knowledge, this is the first qualitative study conducted in Spain to explore the antivaxxers’ knowledge, beliefs, attitudes and barriers regarding vaccination against COVID-19. Our study shows that resistance to vaccination is mainly linked to distrust, conspiracies, and the attribution of causality in the alleged adverse reactions following vaccination.

The results of our study in respect of **distrust** are consistent with those of other studies conducted on antivaxxers [30, 35–38], which also show that distrust towards governments, health professionals and globalist families, like that of Bill Gates, are important determinants of vaccine refusal. Along these same lines, previous studies [39, 40] have also described **conspiracy theories** about plans to control and eliminate the population through vaccination. Hence, conspiracy constitutes the other side of the coin, when it comes to distrust in figures who exercise power or authority over the population. In addition, virus denial [33, 34], low perceived risk [17, 43–47], speed of vaccine development [42, 44, 47–49], dangerous composition [42, 50, 51], doubts as to vaccine efficacy [52–55] and safety [44, 45, 47, 48], lack of information and continuous changes [36, 38, 41, 48, 49], were also factors linked to vaccine refusal in earlier studies. These findings have been obtained, not only with quantitative, survey-based methodology [30, 40, 51], but also with qualitative methodology [42, 44, 48, 49] (like that of our study), which allows for in-depth comprehension of barriers and beliefs associated with anti-vaccination, without the need to administer a questionnaire [56].

According to various authors [57, 58], the opinions of persons with a vaccine-refusal or anti-vaccination attitude are immovable and practically impossible to change, whereas among the vaccine-hesitant there is a higher probability of achieving acceptance of the vaccine. This situation of uncertainty is modifiable and should be targeted by health authorities, so that these subjects come to trust in vaccination, vaccine providers, and health and governance systems [59]. To this end, it could be of use [60, 61]: (1) to spread information about vaccine safety and efficacy across all media, including the same channels as antivaxxers use to spread their myths and conspiracies, although it could not to be accepted; (2) to fight against fake news (3), to identify and counter the tactics used by anti-vaccine groups; and (4), to endorse and support the benefits of vaccines with peer-reviewed, high-quality scientific evidence.

While we feel that these results are relevant, both in the field of COVID-19 vaccination in particular and in that of vaccination in general, we also consider that they may form part of an increasingly broader trend, that of science denial, which covers other aspects of similarly great public health impact, such as climate change, the association between tobacco and lung cancer, and the association between HIV virus and AIDS [62]. On the Internet, this school of thought finds an easy avenue of dissemination and hinders the application of scientific advances [61, 63], something that can have extremely serious consequences for global health. Hence, more co-ordinated efforts are called for by States, international bodies, those in charge of social networks, and the mass media to limit the dissemination of this content matter and counter its influence among the general public [58]. Furthermore, vaccine hesitancy may imply major public health problems, such as difficulty in reaching herd immunity, which will favor disease re-circulation and increase the risk of outbreaks, or the formation of highly susceptible groups to the disease among those who cannot be vaccinated due to specific medical conditions [64].

### Advantages and limitations

This study has advantages and limitations specific to the use of qualitative methodology. Among its advantages is the online nature of the sessions, which made it possible to involve people from all over Spain in the FG. They belong to a group of people traditionally little disposed to collaborate in research studies, and among whom it is difficult to create the necessary atmosphere of trust for expression of their knowledge, beliefs and barriers. Indeed, the interaction that occurs among FG participants does not take place in any other context. Our study confirms that reasons for COVID-19 vaccine refusal are similar to reasons for vaccine refusal more generally. The methodology and design used meet the COREQ criteria for qualitative studies. Among the limitations of the chosen methodology is the impossibility of quantifying the influence of each factor associated with vaccine refusal. Moreover, our results may well not be generalisable to other countries, especially those outside Europe. Sampling was not random: instead, the participants were all volunteers, but even so the number of participants suffices for analysis purposes. All participants in our study were between 30 and 40 years old and most were from urban areas, but we consider that these aspects do not compromise the validity and relevance of our qualitative findings [65]. The presence of the moderators, the dominance effect or groupthink could act as biases in our study. However, we believe that the experience of the moderators and the rigorous and blinded analysis of the results ensured that these inherent characteristics of the study design did not compromise the validity of our results [66, 67]. Due to the fact that the group sessions were conducted telematically, some participants only had an audio link-up.

### Conclusions and implications

Currently, there are a number of different vaccines that are highly effective against diseases with great public health impact, but if a high proportion of the population refuses to be vaccinated, the benefit will be substantially reduced. Our results indicate that conspiracy theories and distrust are two of the main attitudes linked to vaccine refusal, and that social media provide antivaxxers with a broad communication and dissemination highway. In this context, there is a need for governments and health authorities to furnish transparent and genuine information that would boost trust in vaccination and prevent the development of conspiracies and vaccine refusal among the general public. The same channels could be used to counter dangerous anti-vaccine information in particular and anti-science information in general, although it could not to be accepted. All this would result in a benefit for public health worldwide.

### Electronic supplementary material

Below is the link to the electronic supplementary material.

Supplementary Material 1

Supplementary Material 2

## Data Availability

Anonymised interview transcripts are available from corresponding author on request.
