# Supplementary material for: Unmasking vaccine hesitancy and refusal: a deep dive into Anti-vaxxer perspectives on COVID-19 in Spain
Source: BMC Public Health. 2024 Jul 1;24:1751. doi: 10.1186/s12889-024-18864-5 (PMC11218155; doi:10.1186/s12889-024-18864-5)
Supplement: Supplementary file 1 — Supplementary Material 1 [file 12889_2024_18864_MOESM1_ESM.docx]

**Supplementary file 1:** Checklist used at the end of each focus group.

|  | FG1 | FG2 | FG3 | FG4 | FG5 |
| --- | --- | --- | --- | --- | --- |
| COVID-19 vaccine protection | x | x | x | x | x |
| Adverse effects of COVID-19 vaccination | x | x | x | x | x |
| COVID-19 vaccination information sources | x | x | x | x | x |
| Perceived risk to COVID-19 disease | x | x | x | x | x |
| Health professionals and vaccination COVID-19 | x | x | x | x | x |
| Policy makers and vaccination COVID-19 | x | x | x | x | x |
| Pharmaceutical industry and COVID-19 | x | x | x | x | x |
| Acceptance/refusal of other vaccines not COVID-19 | x | x | x | x | x |
