# Supplementary material for: Unmasking vaccine hesitancy and refusal: a deep dive into Anti-vaxxer perspectives on COVID-19 in Spain
Source: BMC Public Health. 2024 Jul 1;24:1751. doi: 10.1186/s12889-024-18864-5 (PMC11218155; doi:10.1186/s12889-024-18864-5)
Supplement: Supplementary file 2 — Supplementary Material 2 [file 12889_2024_18864_MOESM2_ESM.docx]

**Supplementary file 2:** Script used in the focus groups.

**INTRODUCTORY QUESTION**

In Spain, there are still 4.2 million people in the target population who have not been vaccinated against COVID-19.

- What do you think about this situation?

**TRANSITION**

- What do you think about the different ways to achieve immunity against the disease?

- What do you think about the protection of vaccines against infection?

**CORE QUESTIONS**

- What benefits do you think vaccinated people have compared to unvaccinated people?

- What do you think about the severity of the COVID-19 disease?

- Do you think that people who have had problems after COVID-19 vaccination would have avoided them if they had not been vaccinated?

- What do you consider to be the aspect that most concerns the population in relation to vaccines?

- What do you consider to be the most reliable sources of information in relation to the pandemic and the vaccination process? Why?

- How would you assess the information provided by the Ministry of Health and SERGAS (Galician Health Service) about COVID-19 vaccines?

- What do you think about the role of the pharmaceutical industry in COVID-19? What about politicians?

- Do any of the vaccines deserve more credibility/trust than others?

- In view of previous experience with the first and second doses of vaccination, what do you think about the need for the third dose?

The childhood vaccination campaign for children aged 5 to 11 starts throughout Spain.

- What do you think about vaccinating children between 5 and 11 years old?

**FINAL QUESTIONS**

- What do you think about vaccines in general to prevent disease?

***Summary of the points discussed.***

- After all that has been said, is there anything in relation to the pandemic and vaccination that has not been discussed and that you think would need to be addressed?

**REFERENCES**

Rzymski, P., Zeyland, J., Poniedziałek, B., Małecka, I., & Wysocki, J. (2021). The Perception and Attitudes toward COVID-19 Vaccines: A Cross-Sectional Study in Poland. *Vaccines*, *9*(4), 382. https://doi.org/10.3390/vaccines9040382

Kumari, A., Ranjan, P., Chopra, S., Kaur, D., Upadhyay, A. D., Kaur, T., Bhattacharyya, A., Arora, M., Gupta, H., Thrinath, A., Prakash, B., & Vikram, N. K. (2021). Development and validation of a questionnaire to assess knowledge, attitude, practices, and concerns regarding COVID-19 vaccination among the general population. *Diabetes & metabolic syndrome*, *15*(3), 919–925. <https://doi.org/10.1016/j.dsx.2021.04.004>

Mosteiro-Miguéns, D. G., Roca, D. B., Domínguez-Martís, E. M., Vieito-Pérez, N., Álvarez-Padín, P., & Novío, S. (2021). Attitudes and Intentions toward COVID-19 Vaccination among Spanish Adults: A Descriptive Cross-Sectional Study. *Vaccines*, *9*(10), 1135. <https://doi.org/10.3390/vaccines9101135>

Masoud, A. T., Zaazouee, M. S., Elsayed, S. M., Ragab, K. M., Kamal, E. M., Alnasser, Y. T., Assar, A., Nourelden, A. Z., Istatiah, L. J., Abd-Elgawad, M. M., Abdelsattar, A. T., Sofy, A. A., Hegazy, D. G., Femía, V. Z., Mendonça, A. R., Sayed, F. M., Elmoursi, A., Alareidi, A., Abd-Eltawab, A. K., Abdelmonem, M., … KAP-COVIDGLOBAL Investigators (2021). KAP-COVID_GLOBAL_: a multinational survey of the levels and determinants of public knowledge, attitudes and practices towards COVID-19. *BMJ open*, *11*(2), e043971. https://doi.org/10.1136/bmjopen-2020-043971

Kumari, A., Ranjan, P., Chopra, S., Kaur, D., Upadhyay, A. D., Kaur, T., Bhattacharyya, A., Arora, M., Gupta, H., Thrinath, A., Prakash, B., & Vikram, N. K. (2021). Development and validation of a questionnaire to assess knowledge, attitude, practices, and concerns regarding COVID-19 vaccination among the general population. *Diabetes & metabolic syndrome*, *15*(3), 919–925. https://doi.org/10.1016/j.dsx.2021.04.004
